# Plasma proenkephalin A 119–159 on intensive care unit admission is a predictor of organ failure and 30-day mortality

**DOI:** 10.1186/s40635-021-00396-6

**Published:** 2021-07-19

**Authors:** Attila Frigyesi, Lisa Boström, Maria Lengquist, Patrik Johnsson, Oscar H. M. Lundberg, Martin Spångfors, Martin Annborn, Tobias Cronberg, Niklas Nielsen, Helena Levin, Hans Friberg

**Affiliations:** 1 Department of Clinical Sciences, Anaesthesiology and Intensive Care, Lund University, 22185 Lund, Sweden; 2 Skåne University Hospital, Intensive and Perioperative Care, 22185 Lund, Sweden; 3 Skåne University Hospital, Intensive and Perioperative Care, 21428 Malmö, Sweden; 4 Kristianstad Central Hospital, Anaesthesia and Intensive Care, 29185 Kristianstad, Sweden; 5 Helsingborg Hospital, Anaesthesia and Intensive Care, 25187 Helsingborg, Sweden; 6 Skåne University Hospital, Department of Neurology, 22185 Lund, Sweden; 7 Skåne University Hospital, Research and Education, 22185 Lund, Sweden

**Keywords:** Proenkephalin, Biomarker, Intensive care, Mortality, Organ dysfunction

## Abstract

**Background:**

Proenkephalin A 119-159 (penKid) has been suggested as a marker of renal failure and poor outcome. We aimed to investigate the association of penKid on ICU admission with organ dysfunction and mortality in a mixed ICU population. In this retrospective, observational study, admission penKid levels from prospectively collected blood samples of consecutive patients admitted to four Swedish ICUs were analysed. The association of penKid with day-two sequential organ failure assessment (SOFA) scores and 30-day mortality was investigated using (ordinal) logistic regression. The predictive power of penKid for 30-day mortality and dialysis was assessed using the area under the receiver operating characteristic curve (AUC).

**Results:**

Of 1978 included patients, 632 fulfilled the sepsis 3-criteria, 190 had a cardiac arrest, and 157 had experienced trauma. Admission penKid was positively associated with 30-day mortality with an odds ratio of 1.95 (95% confidence interval 1.75–2.18, p < 0.001), and predicted 30-day mortality in the entire ICU population with an AUC of 0.71 (95% confidence interval 0.68–0.73) as well as in the sepsis, cardiac arrest and trauma subgroups (AUCs of 0.61–0.84). Correction for admission plasma creatinine revealed that penKid correlated with neurological dysfunction.

**Conclusion:**

Plasma penKid on ICU admission is associated with day-two organ dysfunction and predictive of 30-day mortality in a mixed ICU-population, as well as in sepsis, cardiac arrest and trauma subgroups. In addition to being a marker of renal dysfunction, plasma penKid is associated with neurologic dysfunction in the entire ICU population, and cardiovascular dysfunction in sepsis.

**Supplementary Information:**

The online version contains supplementary material available at 10.1186/s40635-021-00396-6.

## Introduction

The severity of illness on ICU admission is commonly assessed using the Simplified Acute Physiology Score III (SAPS-3), which includes laboratory measures, physiological parameters, and comorbidities [1, 2]. There is a need to improve and simplify the assessment of the severity of disease and if possible characterise disease phenotypes, e.g. using biomarkers.

Proenkephalin A 119–159 (penKid) is a peptide derived from the same precursor as met- and leu-enkephalins and it has been established as a reliable surrogate marker for the unstable enkephalins. It is stable in plasma for at least 48 h [3]. The gene for penKid is expressed in the central nervous system and in multiple non-neuronal tissues [4], with concentrations of penKid being 100-fold higher in the cerebrospinal fluid compared with serum [3].

Serum penKid has been suggested to be a biomarker for assessment of renal function [5, 6], and an earlier marker of acute kidney injury (AKI) than creatinine [7]. Dépret and colleagues showed in a cohort of critically ill patients that subclinical AKI, defined by elevated penKid > 80 pmol/L in patients without AKI, was associated with mortality similar to that of patients with AKI [8].

Proenkephalin A 119–159 is a small molecule assumed to be freely filtered through the renal glomerulus [9, 10] and does not seem to be directly affected by an inflammatory response, making penKid a specific marker for renal function in inflammatory states such as sepsis [7, 10, 11]. In addition to being a marker of renal function, serum penKid has been proposed to be a marker of brain injury. Yalcin et al. have shown that serum proenkephalin may be used as a predictor of mortality in patients with traumatic brain injury [12]. Associations between elevated penKid levels and functional outcome as well as mortality in patients with traumatic brain injury, ischaemic- and hemorrhagic stroke have also been found [13–16]. A combination of renal dysfunction and brain injury that might explain penKid elevation in critically ill patients has not been explored.

Our hypothesis was that penKid may be valuable for assessment of subsequent organ dysfunction and mortality in critically ill patients. We aimed to investigate the association and prognostic value of admission penKid with organ dysfunction and 30-day mortality in the ICU.

## Materials and methods

### Study design and setting

The study design is a multicenter retrospective analysis and observational study of prospectively collected blood samples. The Standards for Reporting of Diagnostic Accuracy Studies (STARD) guidelines were followed [17].

### Participants

Consecutive patients admitted to any of four mixed surgical and medical ICUs in southern Sweden in 2016 were evaluated for eligibility and in this group all adult patients with valid blood samples were included in the study. The sepsis subgroup was identified using the sepsis-3 criteria. A detailed description of this procedure has been presented elsewhere [18]. The cardiac arrest subgroup was identified using the International Statistical Classification of Diseases and Related Health Problems (ICD) code I46.9 at ICU discharge. The trauma subgroup was identified through polytrauma as the reason for ICU admission. When transfers occurred between the participating ICUs, follow-up data were merged to form cohesive ICU admissions.

### Variables

The third version of the Simplified Acute Physiology Score (SAPS-3) was calculated [1, 2] based on physiological parameters and laboratory findings recorded within 1 h before/after ICU admission.

The Sequential Organ Failure Assessment (SOFA) score was recorded daily during the ICU stay [19]. The day-two SOFA was the first score to be based on a full 24-h period following admission. The total SOFA score was based on the available SOFA subscores.

### Data sources

Background and survival data were extracted from the Patient Administrative System for Intensive Care Units (PASIVA). PASIVA is the portal by which the treating physician and nursing staff submit prospectively collected laboratory and physiological data to the Swedish Intensive Care Registry (SIR). PASIVA is synchronised with the Swedish population register, which contains survival data.

The blood samples were collected in EDTA (ethylenediaminetetraacetic acid) vacutainers at ICU admission, centrifuged to obtain EDTA plasma, aliquoted, frozen, and thereafter stored in the SWECRIT biobank at Region Skåne (registration no. BD-47). For inclusion, the sample had to be collected within 6 h after ICU admission. If the sampling time was missing, samples were included if the time of freezing was within the 6-h time frame. The frozen plasma samples were shipped, and batch analysis of penKid was performed in a blinded fashion on thawed samples using a luminescence immunoassay technique [5] in 2019 at the laboratory of SphingoTec GmbH (Hennigsdorf, Germany). The assay has a limit of detection at 7 pmol/L and a mean interassay coefficient of variation of 5.7% in the measuring range 10.9–686 pmol/L.

No clinical information was available to the performers/readers of the index test. The penKid results were not available to clinicians during patient care.

### Bias

Selection bias was investigated in a comparison of baseline characteristics between included and excluded patients.

### Study size

The study size was based on the number of ICU admissions and valid blood samples during the study period.

### Quantitative variables

In this study, renal failure was defined as a renal SOFA score of $\ge$1 for the purpose of subgroup analyses and $\ge$2 when renal failure was treated as an outcome (AUC).

Reference levels for penKid have been published by Marino et al. 2015 [10] with a mean (± 1 standard deviation) of 47 ± 14 pmol/L, and a 99th percentile of 80 pmol/L.

### Statistical methods

The association between 30-day mortality and penKid levels was analysed using logistic regression. Plasma penKid was included as a base-10 log-transformed z-normalised (linear transformation to 0 mean and standard deviation 1) independent variable. The regression models were evaluated with the Hosmer–Lemeshow goodness-of-fit test with ten groups, and only models resulting in non-significant tests were reported [20]. The association between day-two SOFA scores and penKid levels were analysed using multivariable ordinal logistic regression [21].

To evaluate the additional value of penKid to SAPS-3 in the logistic regression model, the area under the receiver operating characteristic curve (AUC) was calculated [22]. Differences in AUCs were tested with the method of DeLong et al. [23].

By adding admission creatinine as an independent variable in the ordinal logistic regression models, we examined the association of penKid with organ dysfunction independently of renal dysfunction.

## Results

### Participants

Out of 2546 adult ICU admissions, 1978 (78%) had valid blood samples at ICU admission and did not opt out. Of the 1978 admissions, the sepsis subgroup constituted 32% (n = 632), the cardiac arrest subgroup 9.6% (n = 190) and the trauma subgroup 7.9% (n = 157). There was no overlap between the sepsis and the cardiac arrest subgroups (cardiac arrest was an exclusion criterion for sepsis). Of the trauma group 6.5% were identified as having sepsis 3 on admission, and 1.3% of the trauma patients also had a cardiac arrest. See Additional file 1: Figure S1 for a flow chart.

The median time from ICU admission to blood sampling for penKid was 21 min (interquartile range (IQR) 15–40 min). The mean time from ICU admission to the start of day-two SOFA was 17.5 h (standard deviation 6.2 h).

### Descriptive data

The median (IQR) penKid concentration was 85 (54–148) pmol/L for the entire ICU population, 103 (63–194) pmol/L for the sepsis group, 136 (91–215) pmol/L in the cardiac arrest group, and 74 (52–103) pmol/L in the trauma group (Table 1). For the distribution of penKid, see Additional file 1: Figure S2. Fifty-three percent of the ICU patients were above the normal-range 99th percentile of 80 pmol/L.

The female to male ratio, ICU length of stay, ICU mortality, 30-day mortality, morbidity as measured by SAPS-3, median age and variables included in the SAPS-3 score are presented in Table 1.

The patients excluded from the study were slightly younger, had lower mortality, and a shorter ICU length of stay, as presented in Additional file 1: Table S1. The excluded group included fewer sepsis patients but more trauma.

**Table 1:** Descriptive statistics for the whole ICU population, the sepsis-3 subgroup, the cardiac arrest subgroup and the trauma subgroup

	ICU	Sepsis	Cardiac arrest	Trauma
Number of patients	1978	632 (32%)	190 (9.6%)	157 (7.9%)
Women (%)	39	40	27	23
ICU length of stay (days)	1.7 (0.8–3.8)	2.5 (1.1–5.5)	2.4 (0.97–4.3)	1.8 (0.93–4.1)
ICU mortality (%)	11	14	34	4.5
30-day mortality (%)	22	28	54	12
SAPS-3 score	59 (47–71)	66 (57–77)	76 (66–87)	48 (39–59)
Day-one SOFA score	7 (4–10)	8 (6–11)	10 (8–13)	5 (2–7)
Day-two SOFA score (n = 995)	8 (5–10)	8 (6–11)	10 (8–12)	6 (4–9)
CRRT during ICU stay (%)	9.2	15	7.4	0.6
Box I				
Age (years)	66 (54–74)	69 (61–76)	68 (60–76)	55 (33–70)
Comorbidities				
Cancer therapy (%)	4.6	6.3	4.2	0.6
Chronic heart failure (%)	5.5	7.0	10	1.3
Hematological cancer (%)	2.6	5.2	1.0	0
Liver cirrhosis (%)	1.1	1.6	0	0
Cancer (%)	10.4	10.4	7.9	1.3
Vasoactive drugs before ICU (%)	44	47	64	21
Box II				
Surgical status at ICU admission				
No surgery (%)	74	85	94	83
Box III				
GCS	13 (10–15)	13 (10–15)	3 (3–8)	10 (6–15)
Total bilirubin ($\mu$mol/L)	9 (6–15)	10 (7–19)	8 (5–12)	8 (5–12)
Max. temperature ($^\circ$C)	37.0 (36.3–37.6)	37.3 (36.5–38.1)	36.0 (35.5–36.8)	37.0 (36.0–37.5)
Max. creatinine ($\mu$mol/L)	93 (70–145)	119 (79–205)	112 (87–149)	87 (74–111)
Max. heart rate (bpm)	100 (80–118)	107 (90–122)	98 (80–115)	90 (80–110)
Max. leukocyte count ($\times 10^{9}/$L)	13 (8.8–18)	13 (8.4–19)	16 (12–21)	14 (11–19)
Min. pH	7.34 (7.24–7.41)	7.32 (7.20–7.40)	7.19 (7.04–7.3)	7.34 (7.29–7.40)
Min. platelet count ($\times 10^{9}/$L)	221 (161–286)	218 (147–304)	222 (165–270)	212 (171–261)
Min. systolic blood pressure (mmHg)	100 (80–120)	91 (75–115)	85 (68–109)	104 (90–130)
Oxygenation				
Initial respiratory support$^{\dagger }$ (%)	58	59	86	53
$\text {FiO}_{2}$ (%)	50 (40–70)	60 (40–80)	60 (50–80)	45 (40–57)
$\text {PaO}_{2}$ (kPa)	13 (9.8–18)	11 (9.0–15)	13 (11–22)	16 (11–24)
Other				
penKid (pmol/L)	85 (54–148)	103 (63–194)	136 (91–215)	74 (52–103)

If not stated otherwise, values represent medians (interquartile ranges, IQR). Box I–III refers to the subsections of the SAPS-3 scoring system. $\dagger$Respiratory support refers to invasive or non-invasive ventilation within 1 h of admission

ICU: intensive care unit, SAPS-3: simplified acute physiology score III, SOFA: Sequential Organ Failure Assessment, CRRT: continuous renal replacement therapy, GCS: Glasgow coma scale, $\text {FiO}_{2}$: fraction of inspired oxygen, $\text {PaO}_{2}$: arterial partial pressure of oxygen, penKid: Proenkephalin A 119–159

The fraction of missing parameters was mostly low, in the range of 0% for GCS to bilirubin and platelet counts of 7% (Additional file 1: Table S2). Oxygenation parameters on admission were missing from 12% in $\text {FiO}_{2}$ to 49% for $\text {PaO}_{2}$. Day-two SOFA sub-scores were not available in the range of 49 to 53%, almost entirely due to a short ICU length of stay.

**Table 2:** Odds ratios (OR) for Proenkephalin A 119–159 (penKid) from univariate (ordinal) logistic regressions on 30-day mortality and day-two SOFA scores and bivariate (ordinal) logistic regressions on day-two SOFA scores with penKid and admission creatinine as covariates

Univariate	ICU			Sepsis			Cardiac arrest			Trauma		
Outcome	OR	95% CI	p-value	OR	95% CI	p-value	OR	95% CI	p-value	OR	95% CI	p-value
Mortality	**1.95**	1.75–2.18	< 0.001	**1.39**	1.18–1.64	< 0.001	**2.42**	1.63–3.75	< 0.001	**5.53**	2.82–12.62	< 0.001
Cardiovasc. SOFA	**1.57**	1.40–1.77	< 0.001	**1.50**	1.27–1.79	< 0.001	1.01	0.67–1.52	0.97	1.17	0.68–2.02	0.56
Resp. SOFA	1.06	0.95–1.20	0.29	0.89	0.75–1.07	0.22	0.90	0.60–1.36	0.62	**1.76**	1.04–3.00	0.035
Renal SOFA	**5.48**	4.64–6.51	< 0.001	**5.06**	3.99–6.51	< 0.001	**6.57**	3.81–12.13	< 0.001	**8.35**	3.83–20.31	< 0.001
Hepatic SOFA	**1.24**	1.07–1.44	0.0048	**1.27**	1.02–1.58	0.032	0.99	0.53–1.78	0.98	0.91	0.39–1.97	0.82
Neurol. SOFA	**1.49**	1.32–1.67	< 0.001	**1.20**	1.01–1.43	0.038	**1.65**	1.05–2.68	0.035	**2.42**	1.36–4.41	0.0030
Coag. SOFA	1.12	0.99–1.26	0.069	1.12	0.94–1.34	0.22	0.84	0.54–1.28	0.42	1.35	0.79–2.33	0.27

Bivariate	ICU			Sepsis			Cardiac arrest			Trauma		
Outcome	OR	95% CI	p–value	OR	95% CI	p-value	OR	95% CI	p-value	OR	95% CI	p-value
Mortality	**2.48**	2.20–3.03	< 0.001	**1.67**	1.33–2.11	< 0.001	**4.61**	2.66–8.53	< 0.001	**9.41**	3.70–30.57	< 0.001
Cardiovasc. SOFA	**1.64**	1.41–1.92	< 0.001	**1.66**	1.31–2.11	< 0.001	1.57	0.92–2.71	0.099	1.01	0.57–1.80	0.97
Resp. SOFA	1.05	0.90–1.22	0.54	0.91	0.72–1.16	0.46	0.77	0.43–1.36	0.36	1.67	0.96–2.94	0.070
Hepatic SOFA	1.12	0.92–1.36	0.27	1.26	0.94–1.72	0.13	1.34	0.60–3.01	0.47	0.86	0.31–2.27	0.76
Neurol. SOFA	**1.78**	1.51–2.09	< 0.001	**1.37**	1.07–1.76	0.016	**2.13**	1.18–3.97	0.015	1.92	0.94–3.76	0.059
Coag. SOFA	1.09	0.93–1.28	0.28	1.17	0.92–1.50	0.21	0.80	0.44–1.42	0.45	1.55	0.82–3.13	0.20

Significant ORs are shown in bold

CI: confidence interval, SOFA: sequential organ failure assessment

### Unadjusted models

**Fig. 1:**
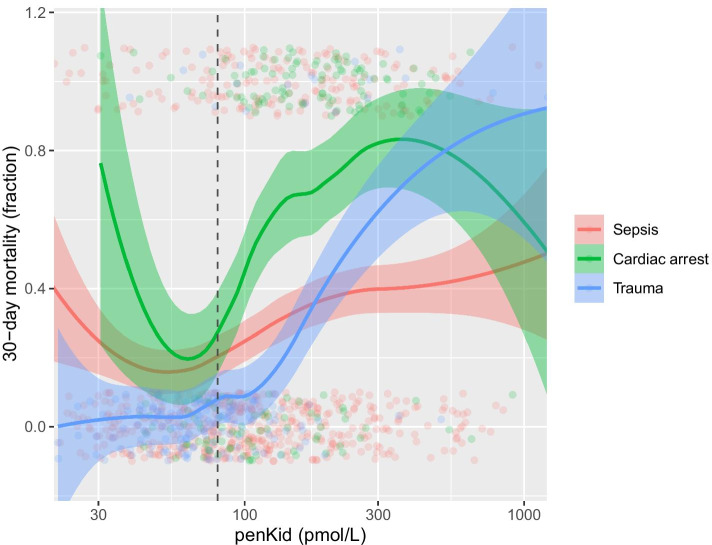
Thirty-day mortality as a function of admission plasma proenkephalin A 119–159 (penKid) concentration in the subgroups sepsis, cardiac arrest and trauma. The vertical line at penKid 80 pmol/L is the upper normal (99th percentile) limit. The continuous lines represent smoothed averages, i.e. the average 30-day mortality in a small neighbourhood of a penKid value. The coloured bands around the smoothed averages indicate 95% confidence bands. Individual patients are represented by dots with jitter added. Thus, y values in the interval (− 0.1, 0.1) represent survivors (0 with added jitter), and y values in the interval (0.9,1.1) represent non-survivors (1 with added jitter)

Thirty-day mortality and day-two SOFA was higher for high penKid levels as presented in Fig. 1 and Additional file 1: Figure S3.

Unadjusted logistic regression models for 30-day mortality and day-two SOFA are presented in Table 2 and Fig. 3. Thirty-day mortality was higher for high penKid levels, with an odds ratio (OR) of 1.95 (95% CI 1.74–2.18, $p < 0.001$) in the entire ICU population, 1.39 (95% CI 1.18–1.64, $p < 0.001$) in the sepsis subgroup, 2.42 (95% CI 1.63–3.75, $p < 0.001$) in the cardiac arrest subgroup, and 5.53 (95% CI 2.82–12.62, $p < 0.001$) in the trauma subgroup.

**Fig. 2:**
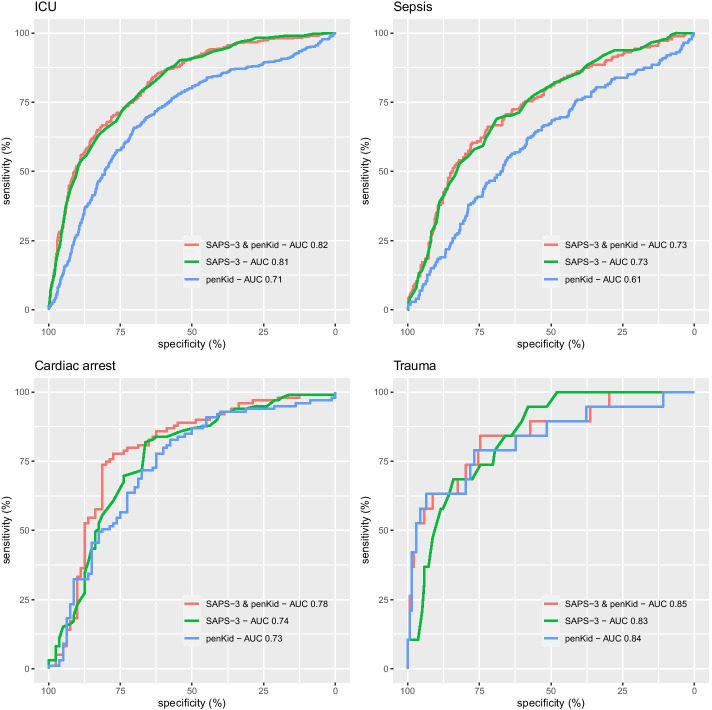
Receiver operating characteristic (ROC) curves and area under curves (AUC) for logistic regression models of 30-day mortality. ICU: intensive care unit, SAPS-3: simplified acute physiology score III, penKid: proenkephalin A 119–159

The AUCs for the logistic regression models on 30-day mortality for penKid was 0.71 (95% CI 0.68–0.73) in the ICU population, 0.61 (95% CI 0.56–0.66) in sepsis, 0.73 (95% CI 0.66–0.80) in cardiac arrest, and 0.84 (95% CI 0.76–0.92) in trauma. See Fig. 2.

**Fig. 3:**
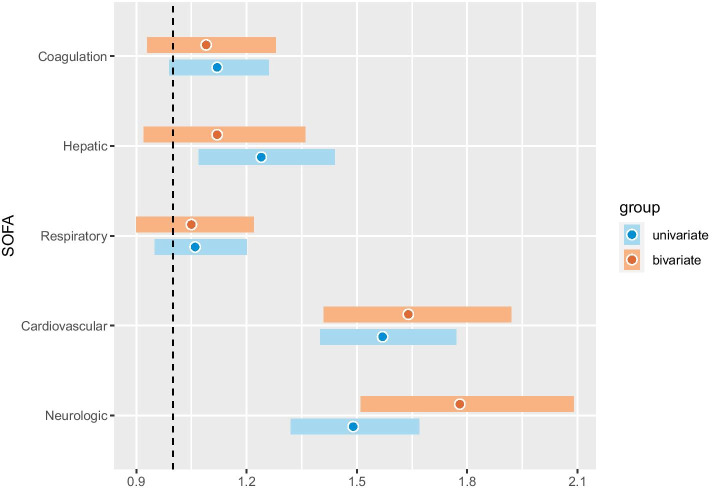
Odds ratios for penKid (proenkephalin A 119–159) in ordinal logistic regression of organ specific SOFA scores in a univariate and a bivariate (including admission creatinine) model

In the ICU population, penKid had a positive association with day-two renal SOFA as well as with cardiovascular, hepatic, and neurological day-two SOFA subscores as presented in Table 2 and Fig. 3. Associations between penKid levels and day-two SOFA subscores in the sepsis, cardiac arrest and trauma subgroups are presented in Table 2.

### SAPS-3 adjusted models

Thirty-day mortality was higher for high penKid levels, independently of SAPS-3, with an OR of 1.32 (95% CI 1.17–1.50, $p < 0.001$) in the entire ICU population, 1.76 (95% CI 1.17–2.73, p = 0.0082) in the cardiac arrest subgroup and 3.64 (95% CI 1.78–8.51, p = 0.0011) in the trauma subgroup. For sepsis, there was no clear association (OR 1.13, 95% CI 0.94–1.36, p = 0.20).

The AUCs for the logistic regression models on 30-day mortality for the ICU population using SAPS-3 was 0.81 (95% CI 0.79–0.83) versus 0.82 (95% CI 0.79–0.84) using SAPS-3 and penKid (p = 0.22). For the sepsis population, the AUC was 0.73 (95% CI 0.69–0.77) using SAPS-3 versus 0.73 (95% CI 0.69–0.77) using SAPS-3 and penKid (p = 0.62). For the cardiac arrest population, the AUC was 0.74 (95% CI 0.67–0.82) using SAPS-3 versus 0.78 (95% CI 0.71–0.85) using SAPS-3 and penKid (p = 0.018). For the trauma population, the AUC was 0.84 (95% CI 0.76–0.92) using SAPS-3 versus 0.85 (95% CI 0.75–0.95) using SAPS-3 and penKid (p = 0.44). See Fig. 3.

### Admission creatinine adjusted models

After correction for admission creatinine, penKid levels were positively associated with cardiovascular and neurological day-two SOFA subscores in the entire ICU population and the sepsis population as presented in Fig. 3 and in Table 2. In the cardiac arrest and the trauma subgroups, there was an association ($\text {OR} > 1.9$) with neurological day-two SOFA. See Table 2.

### Prediction of renal failure and dialysis

The AUC for the logistic regression models on day-two renal dysfunction using admission plasma creatinine as a predictor was 0.91 versus 0.86 using penKid only (p < 0.001). Combining admission plasma creatinine with penKid did not improve the prognostication of day-two renal failure (0.91 vs. 0.86, p = 0.54). The AUC for the logistic regression models on dialysis use for the ICU population using admission plasma creatinine as a predictor was 0.88 versus 0.81 using penKid only ($p < 0.001$). Combining admission plasma creatinine with penKid did not improve the prognostication of dialysis use (0.85 vs. 0.88, p = 1).

## Discussion

Elevated plasma penKid was prognostic of mortality in the entire ICU population, and in all the subgroups. It performed best in the trauma subgroup. In cardiac arrest patients, a combination of penKid and SAPS-3 resulted in significantly better prediction of mortality than SAPS-3 alone.

Several clinical studies have shown that penKid levels correlate with renal function as measured by plasma creatinine or an AKI definition [24]. Our findings corroborate these results by showing that penKid is associated with day-two renal dysfunction as measured by renal SOFA.

In addition, plasma penKid was positively associated with 30-day mortality and day-two cardiovascular, hepatic, and neurological dysfunction as measured by SOFA. To explore the cause of penKid elevation from organ systems other than renal dysfunction in our ICU cohort, we corrected for admission creatinine. This removed a reno-hepatic dysfunction, which led us to hypothesise that these two organ systems were both failing due to a common pathway also leading to penKid elevation. What remained was a component of cardiovascular dysfunction coupled with elevated penKid only seen in the sepsis group, and a third distinct pathway of neurological dysfunction coupled with penKid elevation observable in the entire ICU population and all the subgroups. To summarise, we argue that there is an obvious renal dysfunction component to penKid elevation, a cardiovascular dysfunction component mainly in sepsis, and a neurologic dysfunction component in the entire ICU population. The association between neurologic dysfunction and elevated penKid is intriguing and we propose evaluation of penKid as a potential marker of brain injury. In further studies we aim to analyse the association of penKid with known brain injury biomarkers such as neuron-specific enolase (NSE), and serum neurofilament light (NFL) in cardiac arrest patients [25, 26]. In addition, we aim to further investigate penKid in the subgroup of trauma patients suffering from traumatic brain injury.

In relation to our brain-injury hypothesis, we suggest that a plausible explanation why elevated penKid in patients not meeting the AKI criteria (“subclinical AKI”) was shown to predict mortality after adjustments in the study by Deprét et al. [8], might be due to a degree of brain injury in those patients (of which 4.4% had suffered trauma and 8.8% had suffered out-of-hospital cardiac arrest, i.e. similar to our cohort).

A strength of our study is the well-defined subgroups. The sepsis subgroup, which is often difficult to define, has been carefully characterised and described recently [18]. The cardiac arrest and trauma subgroups are common diagnoses in the ICU and are generally easily identified. Another strength is that we have adjusted for age, comorbidities, and acute severity of illness through the validated SAPS-3 score. After adjustments, our results still hold, showing that penKid provides clinically important information not captured by the SAPS-3 determinants.

### Limitations

The lack of baseline creatinine levels in our study population prevented classification of AKI according to the Kidney Disease Improving Global Outcomes (KDIGO) criteria [27].

The day-two neurological SOFA score for cardiac arrest patients is determined by the fact that most patients are sedated for temperature control on day two [28], and this is therefore not an optimal measure of neurological prognosis.

It should be pointed out that our study was not specifically aimed at investigating the cardiac arrest subgroup and did therefore not correct for well-known cardiac arrest-specific prognostic variables [29].

### Interpretation

To our knowledge, this is the first study investigating the value of admission penKid in a general ICU population as an independent mortality predictor and subsequent organ-dysfunction. Our findings open up a new path of risk assessment for critically ill patients.

### Generalisability

The multicenter approach, with patients from both large university hospital ICUs and regional ICUs, make the results applicable to other high-income hospital settings.

## Conclusion

Plasma penKid on ICU admission is associated with day-two organ dysfunction and predictive of 30-day mortality in a mixed ICU-population, as well as in sepsis, cardiac arrest and trauma subgroups.

## Supplementary Information

**Additional file 1. **Additional tables and figures.

## Data Availability

The datasets used during the current study are available from the corresponding author on request.

## Abbreviations

penKid: Proenkephalin A 119–159
ICU: Intensive care unit
AUC: Area under the receiver operating characteristic curve
AKI: Acute kidney injury
SOFA: Sequential organ failure assessment
SAPS-3: Simplified acute physiology score III
EDTA: Ethylenediaminetetraacetic acid-plasma
IQR: Interquartile range
OR: Odds ratio
GFR: Glomerular filtration rate
TBI: Traumatic brain injury

## References

[1] Metnitz PG, Moreno RP, Almeida E, Jordan B, Bauer P, Campos RA (2005). SAPS 3–From evaluation of the patient to evaluation of the intensive care unit. Part 1: Objectives, methods and cohort description. Intensive Care Med.

[2] Moreno RP, Metnitz PG, Almeida E, Jordan B, Bauer P, Campos RA (2005). SAPS 3–From evaluation of the patient to evaluation of the intensive care unit. Part 2: Development of a prognostic model for hospital mortality at ICU admission. Intensive Care Med.

[3] Ernst A, Köhrle J, Bergmann A (2006). Proenkephalin A 119–159, a stable proenkephalin A precursor fragment identified in human circulation. Peptides..

[4] Denning GM, Ackermann LW, Barna TJ, Armstrong JG, Stoll LL, Weintraub NL (2008). Proenkephalin expression and enkephalin release are widely observed in non-neuronal tissues. Peptides..

[5] Donato LJ, Meeusen JW, Lieske JC, Bergmann D, Sparwaßer A, Jaffe AS (2018). Analytical performance of an immunoassay to measure proenkephalin. Clin Biochem..

[6] Beunders R, van Groenendael R, Leijte GP, Kox M, Pickkers P (2020) Proenkephalin compared to conventional methods to assess kidney function in critically Ill sepsis patients. Shock. 2020;54(3):308–314 10.1097/SHK.0000000000001510 PMC7458088 31977957

[7] Hollinger A, Wittebole X, François B, Pickkers P, Antonelli M, Gayat E (2018). Proenkephalin A 119–159 (Penkid) is an early biomarker of septic acute kidney injury: the kidney in sepsis and septic shock (Kid-SSS) study. Kidney Int Reports..

[8] Dépret F, Hollinger A, Cariou A, Deye N, Vieillard-Baron A, Fournier MC, et al (2020) Incidence and outcome of subclinical acute kidney injury using penkid in critically Ill patients. Am J Respir Crit Care Med. 2020 09;202(6):822–829 10.1164/rccm.201910-1950OC 32516543

[9] Stark M, Danielsson O, Griffiths WJ, Jörnvall H, Johansson J (2001). Peptide repertoire of human cerebrospinal fluid: novel proteolytic fragments of neuroendocrine proteins. J Chromatogr B.

[10] Marino R, Struck J, Hartmann O, Maisel AS, Rehfeldt M, Magrini L (2015). Diagnostic and short-term prognostic utility of plasma pro-enkephalin (pro-ENK) for acute kidney injury in patients admitted with sepsis in the emergency department. J Nephrol.

[11] Kim H, Hur M, Lee S, Marino R, Magrini L, Cardelli P (2017). Proenkephalin, neutrophil gelatinase-associated lipocalin, and estimated glomerular filtration rates in patients with sepsis. Ann Lab Med.

[12] Yalcin A, Baydin A, Tuncel ÖK, Erenler AK, Çokluk C, Güzel M (2017). Diagnostic values of proenkephalin and S100B protein in traumatic brain injury. LaboratoriumsMedizin..

[13] Gao JB, Tang WD, Wang X, Shen J (2014). Prognostic value of neuropeptide proenkephalin A in patients with severe traumatic brain injury. Peptides..

[14] Doehner W, von Haehling S, Suhr J, Ebner N, Schuster A, Nagel E (2012). Elevated plasma levels of neuropeptide proenkephalin A predict mortality and functional outcome in ischemic stroke. J Am College Cardiol.

[15] Chen XL, Yu BJ, Chen MH (2014). Circulating levels of neuropeptide proenkephalin A predict outcome in patients with aneurysmal subarachnoid hemorrhage. Peptides..

[16] Yang XG, An HL, Zhang JM (2014). Neuropeptide proenkephalin A is associated with in-hospital mortality in patients with acute intracerebral hemorrhage. Peptides..

[17] Bossuyt PM, Reitsma JB, Bruns DE, Gatsonis CA, Glasziou PP, Irwig L (2015). STARD 2015: an updated list of essential items for reporting diagnostic accuracy studies. BMJ..

[18] Lengquist M, Lundberg OHM, Spångfors M, Annborn M, Levin H, Friberg H, et al (2020) Sepsis is underreported in Swedish intensive care units: a retrospective observational multicentre study. Acta Anaesthesiol Scand. 2020;64(8):1167–1176 10.1111/aas.13647 32463121

[19] Ferreira FL, Bota DP, Bross A, Mélot C, Vincent JL (2001). Serial evaluation of the SOFA score to predict outcome in critically ill patients. JAMA..

[20] Hosmer DW, Lemeshow S (2000) Applied logistic regression. Wiley series in probability and mathematical statistics. New York: Wiley

[21] Hothorn T, Möst L, Bühlmann P (2018). Most likely transformations. Scandinavian J Stat.

[22] Fawcett T (2015) An introduction to ROC analysis. Pattern Recogn Lett 27(8):861–874

[23] DeLong ER, DeLong DM, Clarke-Pearson DL (2018) Comparing the Areas under two or more correlated receiver operating characteristic curves: a nonparametric approach. Biometrics 3:837 3203132

[24] Khorashadi M, Beunders R, Pickkers P, Legrand M (2020) Proenkephalin: a new biomarker for glomerular filtration rate and acute kidney injury. Nephron. 2020; p. 1–7 10.1159/000509352 PMC7845419 32739920

[25] Freeman WD, Chiota NA (2011). Neuron-specific enolase correlates with other prognostic markers after cardiac arrest. Neurology..

[26] Moseby-Knappe M, Mattsson N, Nielsen N, Zetterberg H, Blennow K, Dankiewicz J, et al (2019) Serum neurofilament light chain for prognosis of outcome after cardiac arrest. JAMA Neurol. 2019 01;76(1):64–71 10.1001/jamaneurol.2018.3223 PMC6440255 30383090

[27] Khwaja A (2012). KDIGO clinical practice guidelines for acute kidney injury. Nephron Clin Pract.

[28] Nolan JP, Soar J, Cariou A, Cronberg T, Moulaert VR, Deakin CD (2015). European Resuscitation Council and European Society of Intensive Care Medicine Guidelines for Post-resuscitation Care 2015: Section 5 of the European Resuscitation Council Guidelines for Resuscitation 2015. Resuscitation..

[29] Søholm H, Hassager C, Lippert F, Winther-Jensen M, Thomsen JH, Friberg H (2015). Factors associated with successful resuscitation after out-of-hospital cardiac arrest and temporal trends in survival and comorbidity. Ann Emerg Med..

