# Supplementary material for: Plasma proenkephalin A 119–159 on intensive care unit admission is a predictor of organ failure and 30-day mortality
Source: Intensive Care Med Exp. 2021 Jul 19;9:36. doi: 10.1186/s40635-021-00396-6 (PMC8286914; doi:10.1186/s40635-021-00396-6)
Supplement: Supplementary file 1 — Additional file 1. Additional tables and figures. [file 40635_2021_396_MOESM1_ESM.pdf]

Table 1: **Descriptive statistics for the included and the excluded groups..** If not stated otherwise, values represent medians (inter quartile ranges, IQR). *ICU: intensive care unit, SAPS-3: simplified acute physiology score III, SOFA: Sequential Organ Failure Assessment.*

	included	excluded	p-value
Number of patients	1978	569	<0.001
Women (%)	39	37	0.39
Age (years)	66 (54-75)	62 (37-73)	<0.001
ICU length of stay (days)	1.7 (0.8-3.8)	1.1 (0.5-2.8)	<0.001
ICU mortality (%)	11	13	0.29
30-day mortality (%)	22	24	0.19
SAPS-3 score	59 (47-71)	54 (42-67)	<0.001
Day-two SOFA score	8 (5-10)	7 (4-9)	0.0081
Sepsis (%)	36	23	<0.001
Cardiac arrest (%)	9.6	8.4	0.39
Trauma (%)	7.9	12	0.0039
No surgery (%)	74	75	0.81

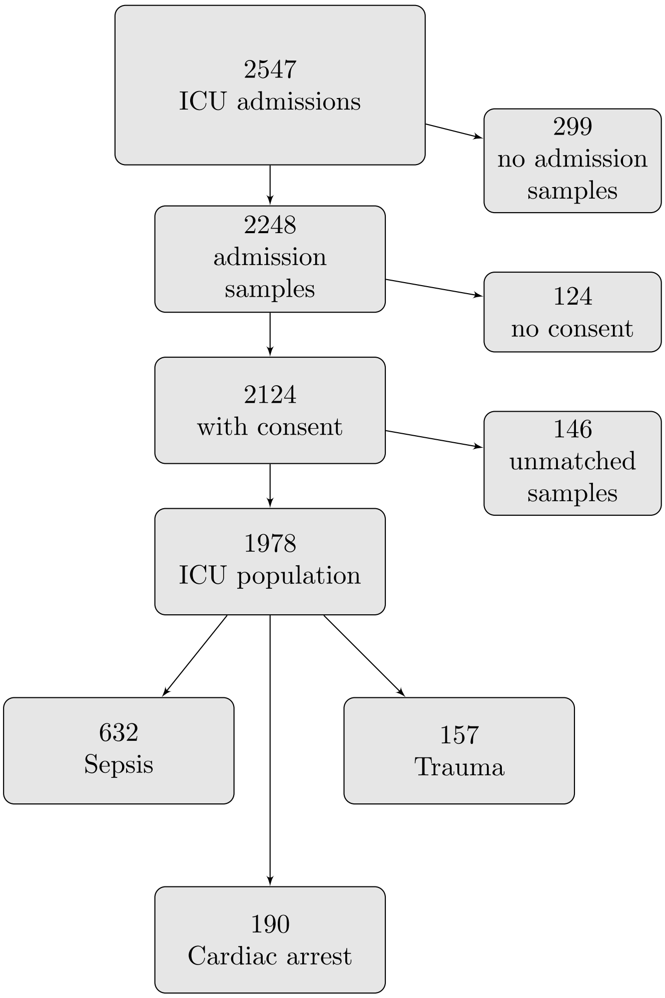

Figure 1: **Flow chart of ICU admissions, admission samples and consent.**

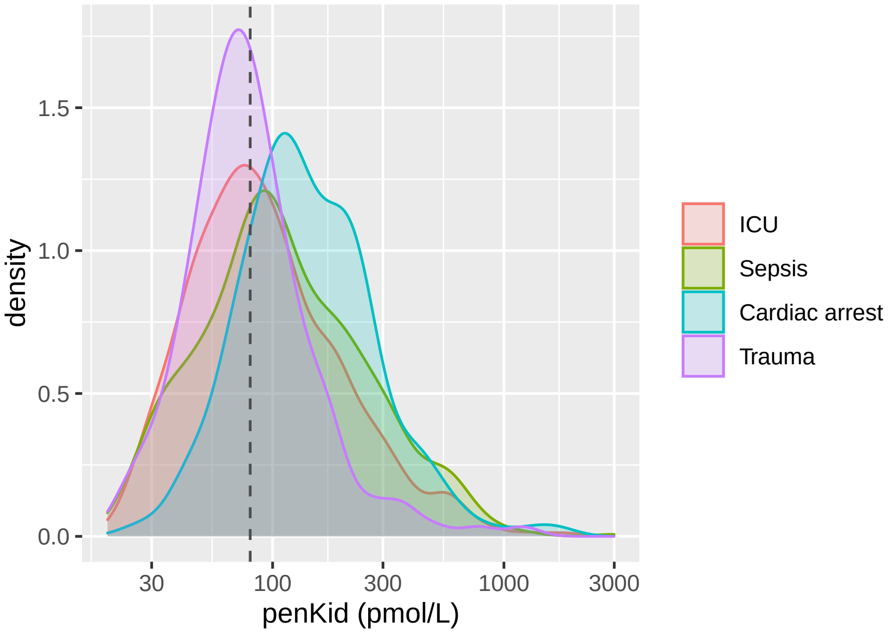

Figure 2: **Distribution of proenkephalin A 119-159 (penKid) levels** in the whole intensive care unit (ICU) population, in the subgroups sepsis, cardiac arrest and trauma. The vertical line at 80 pmol/L indicates the upper normal (99th percentile) limit.

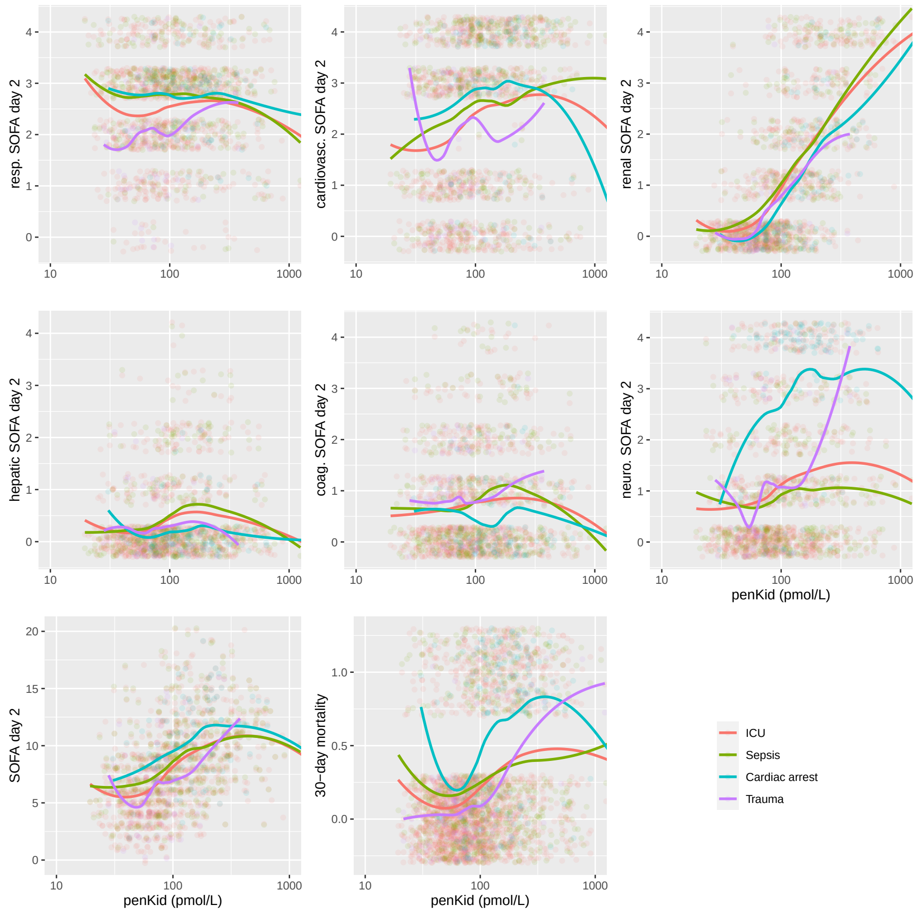

Figure 3: **Organ dysfunction on day two and 30-day mortality** in relation to admission proenkephalin A 119-159 (penKid) level. Individual patients are represented by the dots with added jitter. For mortality, this means that the y-values are in fact 0 and 1, representing 30-day survivors and 30-day non-survivors, respectively. For single-organ SOFA scores, the y-values are the integers from 0 to 4. The continuous lines represent smoothed averages. *SOFA: Sequential Organ Failure Assessment, ICU: intensive care unit.*

Table 2: **Missing data** for the whole ICU population. Variables not reported did not have any missing data. Box III refers to the corresponding section of the SAPS-3 scoring system. Lactate was only reported for patients with sepsis. *SOFA: Sequential Organ Failure Assessment, GCS: Glasgow coma scale,* FiO_2*: fraction of inspired oxygen,* PaO_2*: arterial partial pressure of oxygen.*

	Missing (%)
Day-one SOFA score	16
Day-two total SOFA score	49
Day-two respiratory SOFA score	49
Day-two renal SOFA score	50
Day-two hepatic SOFA score	53
Day-two neurological SOFA score	49
Day-two coagulation SOFA score	51
**Box III**	
GCS	0
Total bilirubin	7
Max. temperature	1
Max. creatinine	5
Max. heart rate (bpm)	0.5
Max. leukocyte count	6
Min. pH	4
Min. platelet count	7
Min. systolic blood pressure	1
Oxygenation	
- Respiratory support	12
- FiO_2	12
- PaO_2 (kPa)	49
Lactate (% of sepsis subgroup)	1
